# Climate Change Increases Reproductive Failure in Magellanic Penguins

**DOI:** 10.1371/journal.pone.0085602

**Published:** 2014-01-29

**Authors:** P. Dee Boersma, Ginger A. Rebstock

**Affiliations:** 1 Department of Biology, University of Washington, Seattle, Washington, United States of America; 2 Wildlife Conservation Society, Bronx, New York, United States of America; Phillip Island Nature Parks, Australia

## Abstract

Climate change is causing more frequent and intense storms, and climate models predict this trend will continue, potentially affecting wildlife populations. Since 1960 the number of days with >20 mm of rain increased near Punta Tombo, Argentina. Between 1983 and 2010 we followed 3496 known-age Magellanic penguin (*Spheniscus magellanicus*) chicks at Punta Tombo to determine how weather impacted their survival. In two years, rain was the most common cause of death killing 50% and 43% of chicks. In 26 years starvation killed the most chicks. Starvation and predation were present in all years. Chicks died in storms in 13 of 28 years and in 16 of 233 storms. Storm mortality was additive; there was no relationship between the number of chicks killed in storms and the numbers that starved (*P* = 0.75) or that were eaten (*P* = 0.39). However, when more chicks died in storms, fewer chicks fledged (*P* = 0.05, *R* ^2^ = 0.14). More chicks died when rainfall was higher and air temperature lower. Most chicks died from storms when they were 9–23 days old; the oldest chick killed in a storm was 41 days old. Storms with heavier rainfall killed older chicks as well as more chicks. Chicks up to 70 days old were killed by heat. Burrow nests mitigated storm mortality (*N* = 1063). The age span of chicks in the colony at any given time increased because the synchrony of egg laying decreased since 1983, lengthening the time when chicks are vulnerable to storms. Climate change that increases the frequency and intensity of storms results in more reproductive failure of Magellanic penguins, a pattern likely to apply to many species breeding in the region. Climate variability has already lowered reproductive success of Magellanic penguins and is likely undermining the resilience of many other species.

## Introduction

Increased frequency of extreme events, such as storms, drought, temperature extremes, and wildfires, associated with climate change, affect many species [1], [2], [3], [4], [5]. Over the past 50 years, more precipitation is coming from heavy rainfall in many areas, and climate models predict the trend will continue [6], [7], even in places where mean precipitation is not predicted to increase [8]. Intense storms kill birds [9], [10], [11], [12] and may affect colonial species more than others [4]. Single storms kill enough seabird chicks to affect reproductive output of colonies [9], [10], [13], [14], recruitment, and population size [15]. Increasing frequency of extreme heat [6] also reduces reproductive success [9], [14], [16], [17], causes adult mortality in birds [5], [9] and increases stress from lack of water [18]. In addition to direct mortality from hyperthermia and hypothermia, extreme weather increases starvation and predation in chicks. Storms may reduce adult foraging efficiency, decreasing the amount, quality, or frequency of food brought to chicks [19], [20], [21], [22]. Storms and heat may also decrease nest attendance by adults, increasing predation on chicks [14], [19], [23], [24]. Seabirds are challenged by indirect effects of climate change, including reduced marine productivity and range shifts of prey species [25]. We investigated whether direct factors, increased storminess and heat, reduce reproductive success in a long-lived seabird, the Magellanic penguin (*Spheniscus magellanicus*).

Many penguin species breed in arid and semi-arid coastal areas, including Antarctica, southern Africa, Peru, the Galapagos Islands, Argentina, and Western Australia. Adult *Spheniscus* penguins have behavioral adaptations to heat [23], [26], [27] and temperate penguins usually nest in shaded sites such as burrows or crevices or under vegetation [28]. Heavy rainfall was infrequent historically in arid areas, and seabird species have not had time to adapt to increasing storm frequency and intensity in the 20^th^ century. On the Antarctic Peninsula, a desert environment that is getting more rain [29], penguin chicks die when their down gets wet and they cannot maintain their body temperature [30].

Near Punta Tombo, Argentina, site of the world’s largest breeding colony of Magellanic penguins [31], rainfall increased and temperature patterns changed between 1960 and 2000 during the austral summer, the penguins’ breeding season. At the Trelew airport weather station (43° 12′ S, 65° 16′ W), about 90 km north of Punta Tombo, precipitation in storms became heavier: the amount of precipitation from wet days (days with at least 1 mm of precipitation), the number of consecutive wet days, the number of days with at least 20 mm of precipitation, and the percentage of total precipitation from days with more than the 99^th^ percentile of rain all increased [32]. Wetter weather was associated with a large-scale spatial pattern of sea-surface temperatures similar to El Niño patterns. Independent of El Niño-Southern Oscillation (ENSO) patterns, storm tracks shifted southward, bringing more precipitation [32]. An increased flow of warmer, moister air from the north accompanied enhanced El Niño-like conditions since 1977 and increased precipitation in the area [33]. At the Trelew airport the daily temperature range increased but there was no significant increase in air temperature. The lowest daily minimum temperature decreased by up to 3°C and the percentage of days with a minimum temperature below the 10^th^ percentile increased [34], [35].

Climate models predict that extreme precipitation in the region will increase in the austral summer by 40%–70% in 2076–2100 compared to 1951–1976 [8] and precipitation events that occurred every 20 years in the late twentieth century are predicted to occur every 10–15 years by 2046 and every 7–15 years by 2081 [7]. Precipitation extremes are expected to increase regardless of whether atmospheric circulation patterns change because warmer air holds more moisture; any particular storm can therefore carry more water [36]. Air temperatures are predicted to increase by 1.5 to 2.5°C in the region over the next century [37], [38].

For most birds, nests help protect eggs and chicks against storms by blocking wind and precipitation and retaining heat [39]. For example, rocks above or around European shag (*Phalacrocorax aristotelis*) nests protected chicks from rain and spray [40]. Fork-tailed Storm-Petrel (*Oceanodroma furcata*) nests in soil were warmer than nests in rocks, leading to higher chick survival [41] and shallow burrows were more likely to flood than deeper burrows [42]. Nests also protect eggs and chicks from overheating in the sun [24], [43]. Ground-nesting passerines in the northern hemisphere orient their nests towards the north at lower latitudes to protect against the heat of the sun and towards the south at higher latitudes to take advantage of the sun’s heat [44].

Chick growth and survival to fledging are strongly linked to food availability in many seabird species [45] and starvation was a major cause of chick mortality at Punta Tombo [46], [47]. Predation on eggs and chicks is an important driver of productivity in many seabirds [48], including Magellanic penguins [24]. We report the major causes of chick mortality at Punta Tombo from 1983 to 2010, including starvation, predation, storms, and heat. We used our field data on chick age and mortality, weather, and nest characteristics to test predictions about chick mortality from storms and to find the best predictor variables of mortality. We used the model to predict mortality rates for chicks for a range of ages and rainfall amounts. We then simulated the effects of decreasing breeding synchrony in the colony on the proportion of chicks vulnerable to a 40-mm rainstorm.

A storm is likely to kill a chick if the chick’s down and skin get wet. A very young chick is likely to be protected by a brooding parent in a well-protected nest and not get wet. Once juvenile plumage covers the skin, it protects an older chick’s skin from getting wet, even if the down is wet. We therefore predicted that chicks of intermediate ages would be more likely to die in a storm than younger or older chicks. We also expected increasing rain and decreasing air temperature to increase chick mortality. We expected burrow nests, nests with more cover, and nests that face north to provide more protection to chicks during storms than bush nests, nests with less cover, and south-facing nests. Nests in burrows maintain a more constant temperature [49], [50] and tend to be better protected from the weather [24] than nests under bushes.

Synchrony of breeding in the colony may affect the proportion of chicks that is vulnerable to a storm and how long a proportion of chicks is vulnerable. Breeding synchrony does not affect an individual chick’s probability of dying, but does affect the proportion of chicks in the colony that is vulnerable to death in a storm. As synchrony decreases, the age span of chicks in the colony increases and the period when some chicks are of a vulnerable age is longer. Magellanic penguins at Punta Tombo bred synchronously with most first eggs laid within about a two week period in the 1980s [47]. We tested whether breeding synchrony decreased and whether laying was less synchronous when laying dates were later. We modeled the consequences of breeding synchrony on the proportion of chicks likely to die in storms on a given day.

## Materials and Methods

We began a long-term study at Punta Tombo, Argentina (44° 03′ S, 65° 13′ W) in 1982, following individual Magellanic penguins and nests [30], [47]. The climate of Punta Tombo is arid, with mean annual precipitation low (<200 mm) but variable [51]. The sparse precipitation when penguins are nesting falls as rain. Penguins arrive at Punta Tombo in September or early October. Females lay two eggs in October, rarely in late September or in November [47], [52], although before egg laying, they usually have 3–4 well-developed follicles with yolk (Boersma unpubl. data). Most eggs hatch between early November and mid-December. Adults take turns foraging, with one parent brooding or guarding the chicks for the first 3–4 weeks [47], [53], after which the parents forage simultaneously and leave the chicks alone. Chicks left alone usually remain in or near their nests rather than forming crèches [53]. Chicks fledge in January or February at 50–100 days of age [53]. We refer to a season by the calendar year that it starts in; 1983 refers to the 1983–1984 season.

We checked all penguin nests from 1983 to 2010 in an area of approximately 7200 m^2^ once or twice a day from mid-September (before eggs are laid) until late February (after most chicks have left the colony). We found all nests used in this study before chicks hatched. On each visit, we recorded the identity of adults, eggs, and chicks in the nest and every 10 days, we weighed and measured chicks. When chicks died or disappeared we recorded the date and determined their cause of death when possible. The number of chicks visited daily ranged from 39 in 2002 to 213 in 1996 (mean = 125, SD = 43, total = 3496). We used subsets of these chicks, where we had relevant data, for the analyses described below.

Magellanic penguins at Punta Tombo nest in burrows that they dig or in shallow depressions under shrubs [24], [54]. We classified 2785 nests as burrow or bush. We classified the quality of each nest according to the percentage of the nest cup covered by earth or foliage: 1 = good (>80% cover), 2 = average (60–79% cover), 3 = poor (<60% cover). Burrow nests usually have more cover than bush nests; 25% of nests were in burrows and 85% of the burrow nests were good (>80% cover) compared to 33% of bush nests. In 1983–1991, 1999, and 2007–2010, we measured the orientation of the main entrance in degrees by pointing a compass from the nest cup toward the entrance (*N* = 1600). The orientation is the direction from which wind or rain enters the nest. For example, if the orientation is 180°, wind from the south blows directly into the nest. We classified orientation, a circular variable, into four cardinal directions: North = 316°–45°, East = 46°–135°, South = 136°–225°, and West = 226°–315°.

Breeding adults and the quality of a nest can change between years (Boersma unpubl. data) even though adults often return and use the same nest in subsequent years [50]. Some nests had one chick and some nests had two chicks. Sibling chicks in the same nest were two days apart in age on average and often did not have the same body condition because parents fed one chick more than the other [55]. Siblings in the same nest did not necessarily share the same fate; in 126 nests where a chick died of exposure and had a sibling, 59 of the siblings died of exposure, 47 died of other causes, and 20 fledged. This is not surprising because a few chicks move from their nests to seek more shelter, the company of other chicks, or to beg for food. Even within a nest, microclimates can vary. Nests and siblings were neither completely independent nor the same. We did not use a repeated-measures analysis; each chick was used once. To account for the lack of independence when chicks shared parents or nests, we grouped on nest ID in logistic regressions on chicks. This procedure reduced the degrees of freedom to the number of individual nest IDs and accounted for the lack of independence among the chicks from the same nest. We also used robust standard errors in the regressions [56].

At Punta Tombo, we collected weather data daily, usually before 0800 h. We recorded precipitation (±0.1 mm) using a manually-emptied plastic rain gauge, and minimum and maximum temperatures (±1°C) using a minimum-maximum recording thermometer for the previous 24 hours. We defined a storm as a period of consecutive days with measurable rain, ranging from one to six days (165 of 233 storms lasted one day and 50 lasted 2 days; only 18 lasted more than 2 days). For storms lasting more than one day, we added the rain for the consecutive days. We defined the low temperature as the lowest daily minimum temperature from the start of the storm to the day following the end of the rain. The temperature often dropped after the rain ended.

We did necropsies on dead adults and chicks opportunistically [57]. When we found a dead chick we determined a cause of death when possible. We assigned predation as the cause of death if we found a dead chick with bite marks or saw signs that a predator had gotten into the nest, such as fresh digging by an armadillo (*Chaetophractus villosus*). If a chick disappeared before 10 January, we assumed a predator took it. Kelp gulls (*Larus dominicanus*) and Antarctic skuas (*Stercorarius antarcticus antarcticus*) are the main predators of young chicks and they usually do not leave evidence of their predation. Other predators include skunks (*Conepatus humboldti*), foxes (*Lycalopex culpaeus*, *L. griseus*), weasels (*Galictis cuja*), and cats (*Leopardus geoffroyi*). Predators may not leave tracks or their tracks may be covered by penguin tracks. Because the chick may have died and been scavenged, we likely overestimated predation and underestimated other causes of death, especially starvation. Chicks that lost weight between measurements, or were very small or skinny for their age, or had empty stomachs and no body fat when found dead, we classified as starved. Additional evidence for starvation was the failure of adults to changeover at the nest for several days when chicks were less than two weeks old. We assumed that a chick found dead following a storm died of exposure during the storm if it had no sign of injury and was a healthy weight. We also assumed that a dead chick with wet down died of exposure even if its weight was low. We found many wet chicks outside of the nest cup and we classified them as storm deaths. We did necropsies on 15 chicks that died in a storm on 17 December 2009 and six chicks that died in a storm on 24 December 2012 to determine whether their stomachs contained food, so we could rule out starvation as the cause of death. Similarly, we assigned heat as the cause of death if a chick looked healthy but died on a hot day (>37°C). Penguins that are overheated often lie with both legs extended to dissipate body heat through unfeathered skin, and we sometimes found dead chicks in that posture following hot days (>30°C) or dead in the shade next to their nests with their bills open indicating they were panting when they died. We likely underestimated deaths from heat because they are hard to determine unless the chick was seen panting before it died. We lumped several minor causes of death into an “other” category, including crushed or pecked by an adult, burrow cave-in, chick died hatching, and possible toxic algae blooms (chicks fed toxic fish or squid). In some cases, we could not determine cause of death and assigned a code of “unknown”. If a chick was not found dead and weighed at least 1800 g after 10 January, we assumed it fledged.

If we found a chick on the first check of the day, we assumed the chick hatched the previous day; if we found the chick on the second check of the day, we assumed it hatched that day. In a few cases (*N* = 151 of 3496) there was an interval of two or three days between nest checks. In those cases, we assumed the chick hatched on the day of the last check before we found it. We included these chicks because they increased the sample size of chicks that died of exposure during storms by 9% but we may have overestimated their ages by a few days. Including or excluding them did not alter our conclusions.

We assigned a storm and an age to each chick that was alive during a storm (*N* = 2482; no storms occurred while 1014 chicks were alive). If a chick died during or immediately after a storm, the chick’s age was its age on the date of the storm that killed it (the first date of a multi-day storm). If the chick did not die in a storm, we used the chick’s age on the date of a randomly-assigned storm that occurred when the chick was known to be alive. These chicks may have fledged or died later of a cause other than a storm. The randomly-assigned storm may or may not have killed other chicks. The number of storms per year ranged from zero in 1988 to 18 in 2005 (mean = 8.3 storms/yr, total storms = 233). Each chick, whether it survived or died in a storm, had an age on a storm date, and a rainfall amount and low temperature from that storm. There were no storms during the chick-rearing period in 1988 so we excluded that season from storm analyses. For each chick that did not die in a storm, we calculated the age when it died, or its age when we last saw it, and whether it disappeared or likely fledged.

### Statistical Analyses

We tested whether chick age, amount of rain, or low temperature affected a chick’s probability of dying during a storm using our 28 years of data with multiple logistic regressions. The response variable was whether each chick died or survived a storm and we standardized age, precipitation, and low temperature so that each had a mean of zero and a standard deviation of one. When the explanatory variables are standardized, the regression coefficients reflect their relative importance [58]. To allow a peak in mortality at intermediate ages in the regression, we included chick-age squared but also tested models without age squared. We included all 2-way interactions except age × age squared because we did not want to include a cubic fit for age which is unlikely to have biological meaning. We also tested the two 3-way interactions that did not include age and age squared: age × rain × low temperature and age squared × rain × low temperature. We excluded the 4-way interaction because it included both age and age squared. We knew age, precipitation, and temperature for 2482 chicks (590 nests) for 1983–2010. We used AIC [59] to select the best regression model. All regressions were run in Stata 9.2 (StataCorp LP, College Station, Texas, USA).

After selecting the best model using chick age and weather variables, we added nest characteristics (type, quality, and orientation) for the subset of 377 nests with 1063 chicks where we had all data on nest characteristics. We again selected the best regression model using AIC.

To determine if storm deaths were additive to other sources of mortality, we regressed the number of chicks that fledged each year on the number of chicks that died in storms. A negative relationship would indicate additive mortality. We also regressed the number of chicks that starved and the number eaten on the number that died in storms. Negative relationships would indicate that storm deaths were compensated by lower starvation or predation rates.

We calculated the 5^th^ and 95^th^ percentile of laying dates of first eggs (when 90% of first eggs were laid) for each year from 1983 to 2010 (*N* = 8033 clutches). We used the number of days between the dates of the 5^th^ and 95^th^ percentiles as an index of laying synchrony. We regressed this index on year to determine if laying synchrony had increased (smaller range of days) or decreased (larger range of days) over time. We removed the trends from the time series of lay dates and laying interval by calculating the residuals from their regressions on year. We regressed the residuals on each other to determine if laying is less synchronous in late years independent of trends over time. We weighed 13 to 213 females (mean = 47, total = 1033) in the second half of September (near arrival dates to the colony) in 22 years and regressed median lay date on the mean weight to determine if females were in poorer body condition when eggs were laid late. There was no trend in mean weight over time (*F*_1,20_ = 1.3, *P* = 0.27).

### Predicted Probability of Death

We estimated a chick’s probability of dying during a storm using the best regression model for chicks in burrow nests and for chicks in bush nests. We calculated the predicted probabilities for chicks in each nest type from 0 to 50 days of age for seven values of precipitation (10, 15, 20, 25, 40, 45, and 55 mm). We calculated age squared and the age-precipitation and age-squared-precipitation interaction terms using the 51 ages and seven precipitation values. We held low temperature and all interactions with low temperature at their mean values (zero because low temperature was standardized).

We simulated the effects of breeding synchrony on chick mortality in storms. We simulated the proportion of chicks likely to die in a storm on a given day by the hatching spread: for 13 days (the mean for 1983–1986) and 27 days (the predicted value for the early 2080s, based on an increase of 0.15 days per year; see results). We assumed a normal distribution of chick hatching dates for each breeding-synchrony group, with the midpoint as the mean. We drew 10,000 random numbers representing chicks for each hatching interval. We binned the numbers (chicks) into 13 categories (days) for the 13-day interval and 27 categories (days) for the 27-day interval. The number in each bin represented the number of chicks hatched on that day, e.g., if the first bin contained 44 numbers then 44 chicks hatched on the first day. Each day for the first 10 days, 3% of chicks were removed from the matrix, and 0.5% of chicks were removed each day thereafter, representing mortality from all non-storm sources based on the mortality we found in the field [46]. We multiplied the number of chicks remaining in each hatch-day bin by the probability that a chick of that age in a bush nest and in a burrow nest would die in a storm with 40 mm of rain. We calculated the number of chicks likely to be killed on each day as the sum of the number of chicks likely to die in each cohort that had hatched by that day (i.e., chicks hatched on day 2 or later were not counted on day 1, etc.). Finally, we converted the number of chicks likely to be killed to a percent.

### Ethics Statement

Work was done on private land with permission from the owners, the La Regina family, and on the Chubut Provincial reserve of Punta Tombo with permits issued by provincial authorities in the departments of Turismo and Fauna. Permits were also issued by the IACUC of the University of Washington (# 2213-02).

## Results

### Causes of Death

**Figure 1:**
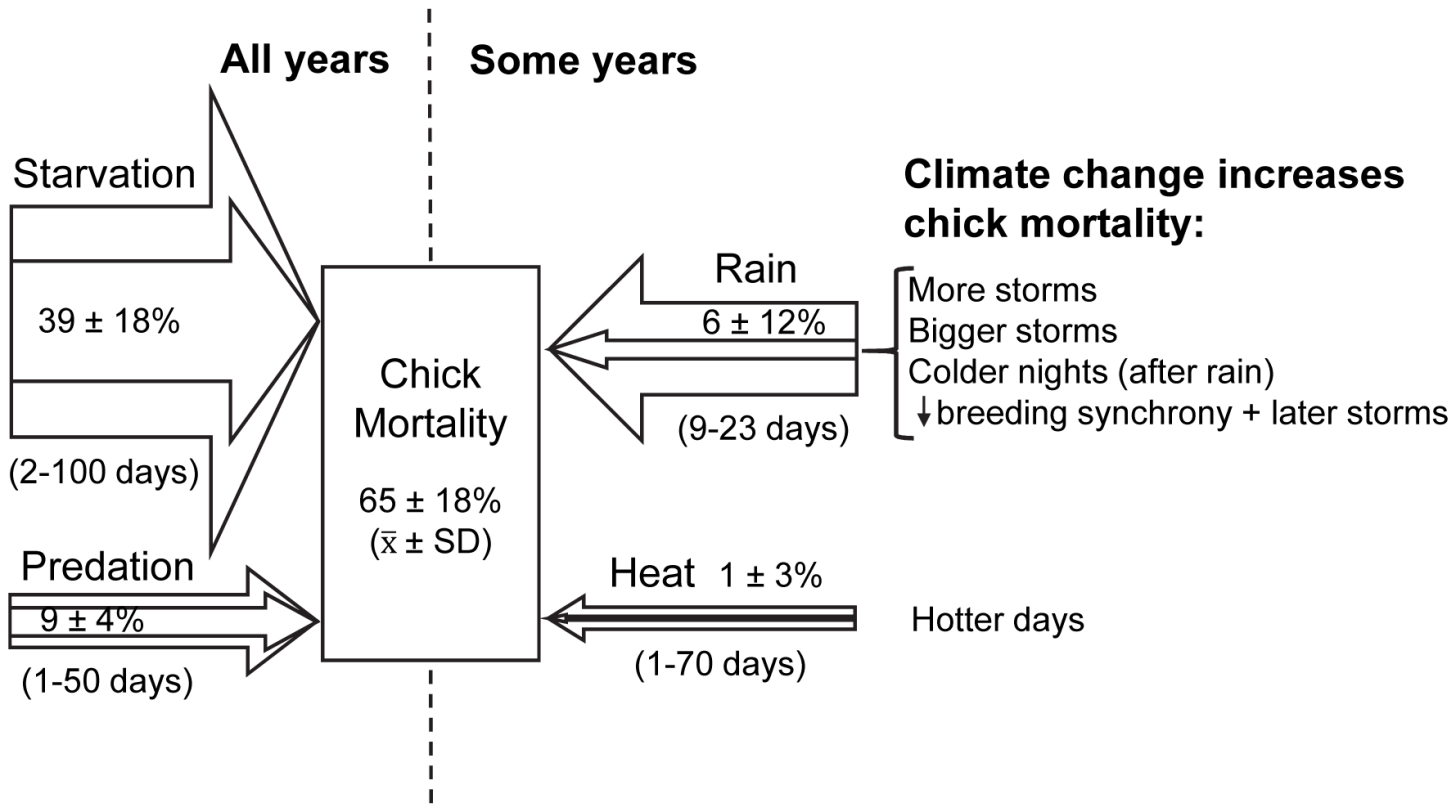
Schematic diagram showing causes of death of Magellanic penguin chicks. Starvation and predation killed chicks in all years; rain and heat killed chicks in some years. The overall mean percentages of chicks killed by rain and heat are smaller than the means for starvation and predation, but the variability is higher for rain and heat than for starvation and predation. In 2 years, rain killed more chicks than starvation and predation. Height of the inner arrows is proportional to the means. Height of the outer arrows is proportional to the mean ±1 standard deviation. Days in parentheses under each arrow refer to the range of ages at which a chick is most vulnerable to that cause of death. Means in the arrows do not total the overall mean mortality rate in the rectangle because the overall mean includes unknown and other causes of death. The list on the right indicates ways that climate change will increase the mean and variability of chick mortality by rain and heat. *N* = 28 years, 3496 chicks.

**Figure 2:**
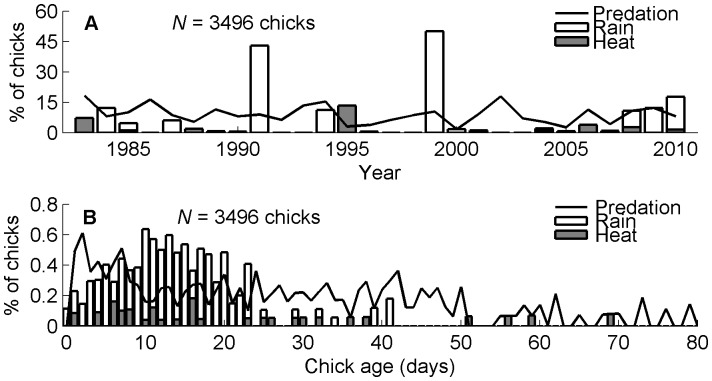
Percentages of Magellanic penguin chicks that died from predation, rain, and heat. (A) Percentages of chicks by year. Predation (solid line) killed chicks in all years; rain (white bars), and heat (gray bars) killed chicks in some years and were sometimes important causes of death. *N* = 28 years, 3496 chicks. Percentages do not sum to 100 because other causes of death are not shown. (B) By chick age (days). The number of chicks that died from predation (solid line), rain (white bars), and heat (gray bars) divided by the total number of chicks that reached each age. Each chick was counted in each age until that chick died or disappeared. The sample size decreases with age: for 0 days of age, *N* = 3496 chicks; for 80 days of age, *N* = 625 chicks.

Chicks starved and were eaten by predators in all years. Rain and heat, climate factors, killed chicks in some years (Fig. 1). Starvation killed 12–86% of chicks (mean = 39%, *N* = 28 years, 3496 chicks) and predators killed 2–18% of chicks (mean = 9%). Starvation and predation killed the most chicks in most years, but deaths from rain and heat were more variable and more important than other causes of death in a few years. The mean deaths from rain (6%, percentage based on 3496 chicks whether or not a rainstorm occurred when each chick was alive) and heat (1%) were lower than the means from starvation and predation, but the coefficients of variation for rain and heat were 4 to 7 times higher than the CVs for starvation and predation (starvation CV = 0.46, predation = 0.44, rain = 2.0, heat = 3.0). In 1999 rain killed as many chicks as all other causes of death combined and in 1991 rain killed as many chicks as starvation and predation combined (Fig. 2a).

**Figure 3:**
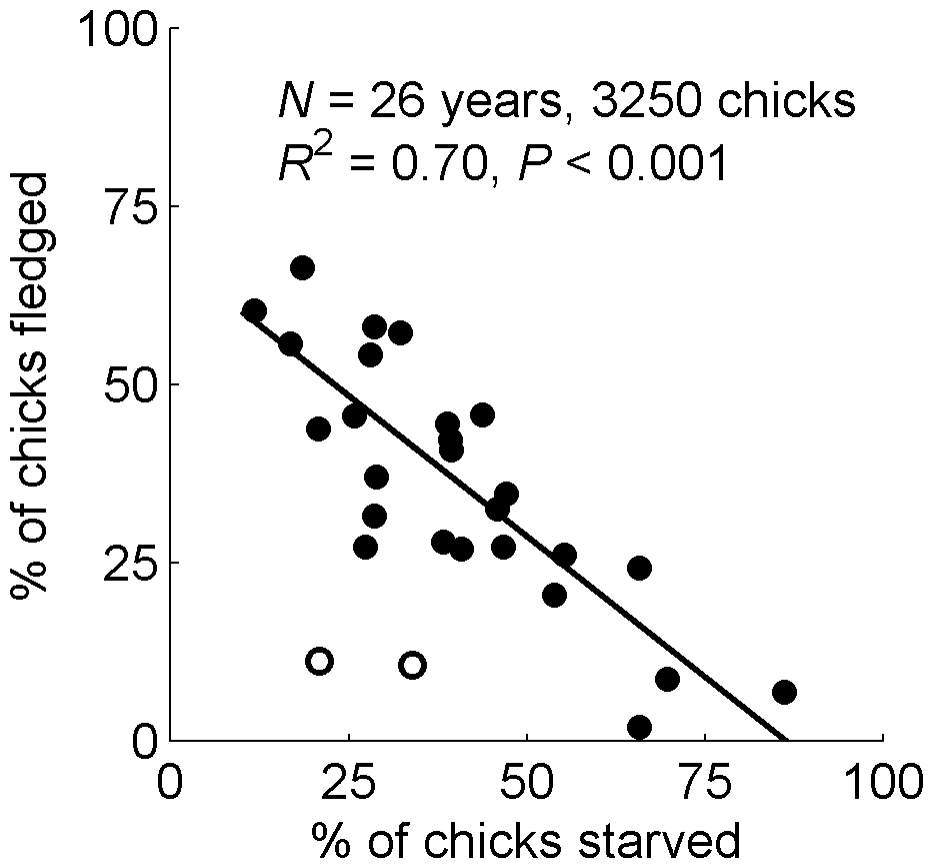
Relationship between starvation and fledging in Magellanic penguin chicks. When a greater percentage of Magellanic penguin chicks starved, a lower percentage fledged (*F*_1,24_ = 56.4, *P*<0.001, *R* ^2^ = 0.70, *N* = 26 years, 3250 chicks). The 2 open circles represent 1991 and 1999, when rain killed >40% of chicks each year, and were not included in the regression.

Starvation strongly affected reproductive success. When a higher percentage of chicks starved, a lower percentage fledged (Fig. 3; *F*_1,24_ = 56.4, *P*<0.0001, *R* ^2^ = 0.70 excluding 1991 and 1999). Storms also affected reproductive success, and deaths from rainfall and heat, climate factors, were additive to deaths from starvation and predation. Significantly fewer chicks fledged when more chicks died in rainstorms (*F*_1,26_ = 4.3, *P* = 0.05, *R* ^2^ = 0.14). However, there was no relationship between the number of chicks that starved and the number of chicks that died in rainstorms (*F*_1,26_ = 0.1, *P* = 0.75), or between the number of chicks predated and the number that died in rainstorms (*F*_1,26_ = 0.8, *P* = 0.39). There were fewer deaths from heat than from rainstorms and no relationship between the number of chicks that died from heat and the number of chicks that starved (*F*_1,26_ = 0.3, *P* = 0.61), the number of chicks predated (*F*_1,26_ = 1.1, *P* = 0.30), or the number that fledged (*F*_1,26_ = 0.8, *P* = 0.38).

We removed one or both eggs in 14 nests within 24–48 hours of laying in 1986 and only one female relaid an egg. Of 471 nests in which an egg was lost naturally soon after laying, 5 females relaid an egg. Of the six females (1%) that relaid eggs, we weighed three and all were heavier (4.1–5.1 kg, but not significantly heavier) than the mean weight of females weighed between eggs in all years (3.8±0.36 kg, *N* = 775).

Chicks were most likely to starve when they were between 5 and 9 days of age. Although starvation decreased with age some chicks starved at 100 days of age. Chicks were most likely to be eaten or disappear between 1 and 7 days of age and few chicks were predated after they were older than 50 days of age (Fig. 2b). Chicks were most likely to die in rainstorms when they were between 9 and 23 days of age. The oldest chicks that died of exposure were 41 days old. Seven chicks older than 30 days died in storms, all in nests that had running flood water. Half (52%) of the chicks that died in storms were younger than 13 days old. A chick’s probability of dying in a storm declined rapidly when it was 20 to 30 days old, the age when chicks are often left alone because both parents are foraging. Chicks up to 70 days of age died from heat but age was a poor predictor of whether a chick might die from heat. Heat likewise kills juveniles and adults when they cannot find sufficient shade and cannot retreat to the water (Boersma unpubl. data).

### Mortality from Rainstorms and Heat

From 1983–2010, 206 known-aged chicks (8% of 2482 chicks alive during a storm) died in or after storms. No chicks died in 217 of the 233 storms (93%). Sixteen storms (14 in December, 2 in November) in 13 of the 28 years killed between <1% and 70% of the chicks. One chick died in a storm with only 1.2 mm of rain (with a low temperature of 3°C), but 97.6% of chicks killed experienced storms with at least 10 mm of rain. Of 21 chicks we necropsied immediately after storms all but one chick had food in the stomach. The one chick with an empty stomach hatched the day before the storm. Six chicks had full stomachs, 11 had 30–450 g of food, two had 5–10 g of fish, and one had some food that we didn’t weigh. None had any sign of injury or could likely have died from a cause other than the storm.

Most chicks that died in storms were attended by at least one parent. In 1999, a storm with 43.5 mm of rain (44% of mean rainfall during the breeding season, October – February) killed 67 chicks. A parent was present at all but one of the nests before and after the storm. At the one nest where an adult was not present after the storm, the nest was flooded, and the adult penguin was 2 meters away in another nest.

The number of chicks that died in storms each season did not increase over time because storms are episodic and vary in timing and rainfall. However, consistent with the increased precipitation in the region [32], [33], we found storm frequency from early November through February increased between 1983 and 2010 at Punta Tombo (*N* = 28, Spearman’s *ρ* = 0.67, *P*<0.001). The number of storms during the first two weeks of December, when all chicks are under 30 days of age and most vulnerable to storm death, also increased between 1983 and 2010 (*N* = 28, Spearman’s *ρ* = 0.43, *P* = 0.02). Moreover, for the most recent three years, 2008 to 2010, storms killed more than 5% of chicks each year (Fig. 2a).

Precipitation ranged from 0.1 to 142 mm per storm with a mean of 98.8 (±51.3) mm of rain during the breeding season and low temperatures ranged from 1° to 18°C. Storms that killed chicks averaged 29.4±36.1 mm of rain (*N* = 16 storms). Storms when no chicks died averaged 7.3±13.8 mm (*N* = 217 storms; *t*_231_ = 5.3, *P*<0.0001). Low temperatures during storms that killed chicks averaged 7.9±3.1°C. Low temperatures during storms that did not kill chicks averaged 9.7±3.3°C (*t*_229_ = 2.1, *P* = 0.04).

Extreme temperatures, whether low or high, can kill chicks when they are not protected by a parent, or are in nests with little shade or protection. Five chicks died of hypothermia, but not during a rainstorm. All five were alone or not brooded by the adult that was present. Forty chicks died from heat, although this is probably an underestimate because of the difficulty of determining death from heat. Air temperatures of 30°C and higher killed chicks, but 75% of the chicks died on days when the temperature was 34°C or higher. The highest temperature recorded when chicks died was 43°C. Like storms, temperature extremes are not predictable.

### Regression Model Results

**Figure 4:**
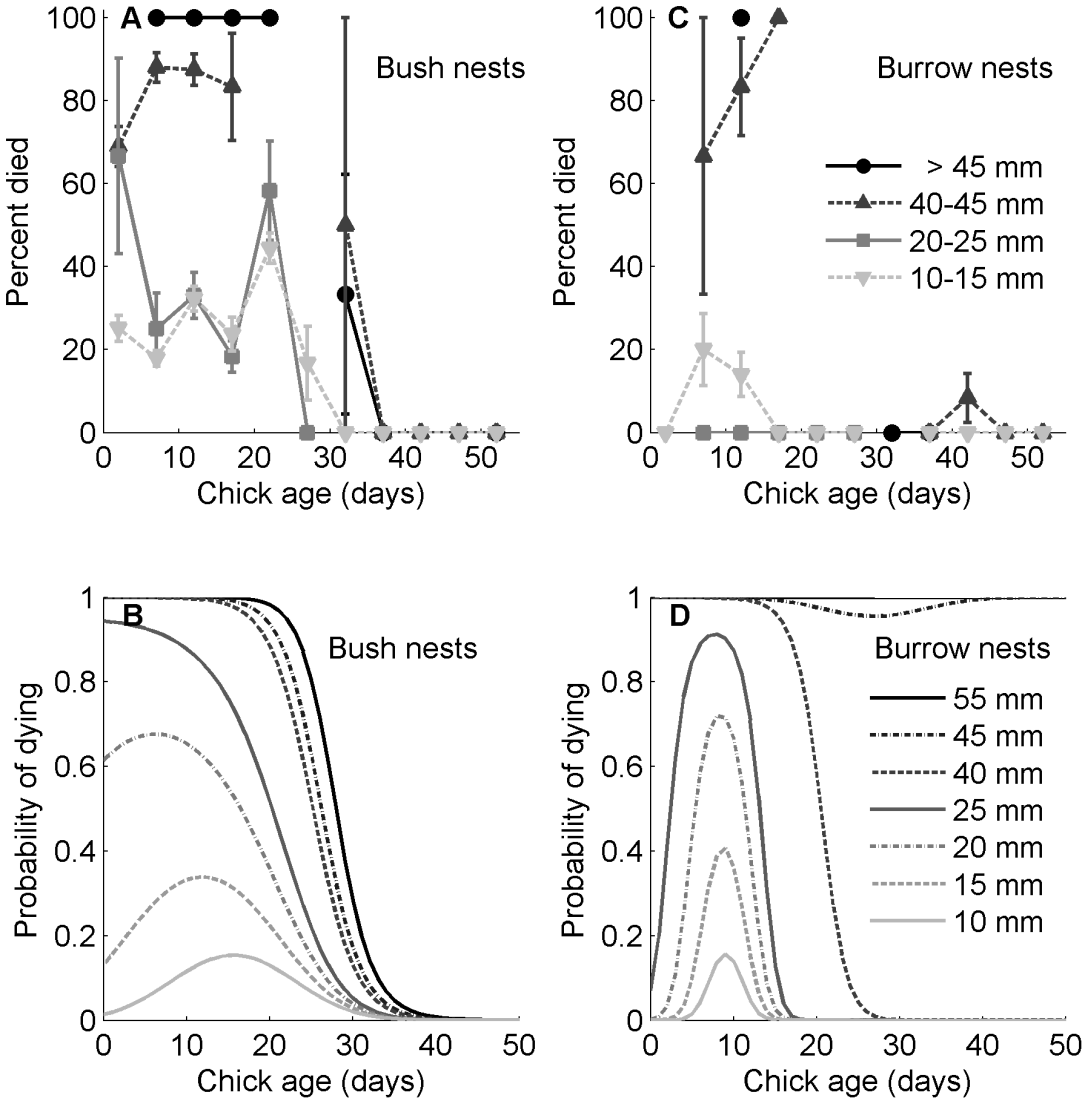
Storm mortality (observed and predicted) in Magellanic penguin chicks, by nest type, age, and rainfall. Mortality increased with higher rainfall, but depended nonlinearly on chick age. *N* = 28 years, 2482 chicks alive during a storm; 206 died of exposure. Top panels: A & C are the observed percentages of chicks that died from age 0 to 55 days for 4 levels of rain. (A) Bush nests: 24 of 44 chicks died in 4 storms with >45 mm rain. 59 of 138 chicks died in 4 storms with 40–45 mm rain. 20 of 47 chicks died in 3 storms with 20–25 mm rain. 40 of 215 chicks died in 14 storms with 10–15 mm rain. (C) Burrow nests: 4 of 10 chicks died in 4 storms with >45 mm rain. 7 of 28 chicks died in 4 storms with 40–45 mm rain. 0 of 16 chicks died in 3 storms with 20–25 mm rain. 6 of 60 chicks died in 14 storms with 10–15 mm rain. Bottom panels: B & D are the predicted probabilities of a chick dying in a storm. Probabilities were calculated from the best logistic regression model (lowest AIC) with age, precipitation, and low temperature standardized plus age squared and interactions. Low temperature and its interactions were held constant for these simulations. (B) Bush nests. (D) Burrow nests.

**Figure 5:**
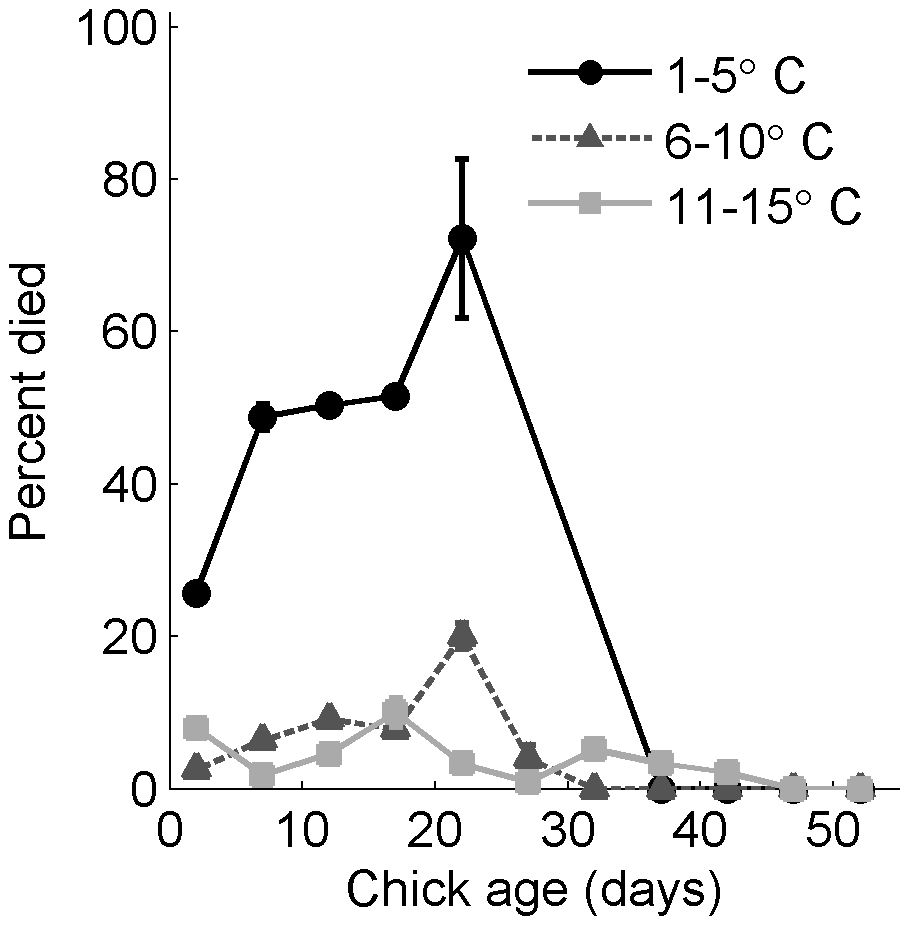
Magellanic penguin chick mortality from storms, by age and low temperature. Mortality increased with lower minimum temperatures, but depended nonlinearly on chick age. *N* = 28 years, 2482 chicks alive during a storm; 206 died of exposure. Percent of chicks that died from age 0 to 55 days for 3 categories of low temperature for bush and burrow nests combined. 127 of 360 chicks died in 27 storms with low temperature of 1–5°C. 52 of 1058 chicks died in 97 storms with low temperature of 6–10°C. 27 of 1009 chicks died in 100 storms with low temperature of 11–15°C. No chicks died in 7 storms with low temperature >15°C (not shown).

Chick age, age squared, and their interactions with rain were the best predictors of chick death in the regression model (Tables 1, 2). Age squared was important, alone and in interactions, demonstrating the nonlinear relationship between mortality and chick age. Models without age squared or its interactions had ΔAIC values >28 and AIC weights <0.001. The probability of dying increased as the amount of rain increased (Fig. 4) and the minimum temperature decreased (Fig. 5), but depended nonlinearly on age and on the interactions between age squared, rain, and low temperature. Pseudo-R^2^ (the proportional difference between the log likelihood of the model and the log likelihood of the intercept-only model) was 0.57 for the best model.

**Table 1:** Predictor variables and model AIC values for the probability that a Magellanic penguin chick died in a storm at Punta Tombo, Argentina, 1983–2010.

Predictor variables	*k*	AIC	ΔAIC	LL	AIC weight
a, a^2^, r, a*r, a^2^*r, a^2^*l, a^2^*r*l (no Low, no interactions between Low and Age or Rain,3-way interaction uses Age^2^)	8	622.79	0	−303.4	0.47
a, a^2^, r, l, a*r, a^2^*r, a^2^*l, a^2^*r*l (includes Low, but no interactions betweenLow and Age or Rain, 3-way interaction uses Age^2^)	9	624.03	1.23	−303.0	0.25
a, a^2^, r, l, a*r, a*l, a^2^*r, a^2^*l, a^2^*r*l (no Rain-Low interaction, 3-way interaction uses Age^2^)	10	625.28	2.48	−302.6	0.14
a, a^2^, r, a*r, a*l, r*l, a^2^*r, a^2^*l, a^2^*r*l (no Low, 3-way interaction uses Age^2^)	10	626.77	3.98	−303.4	0.06
a, a^2^, r, l, a*r, a*l, r*l, a^2^*r, a^2^*l, a^2^*r*l (3-way interaction uses Age^2^)	11	627.03	4.23	−302.5	0.06
a, a^2^, r, l, a*r, a*l, r*l, a^2^*r, a^2^*l, a*r*l, a^2^*r*l (full model)	12	629.03	6.23	−302.5	0.02
a, a^2^, r, l, a*r, a*l, r*l, a^2^*r, a^2^*l, a*r*l (3-way interaction uses Age)	11	635.45	12.65	−306.7	<0.001
a, a^2^, r, l, a*r, a*l, r*l, a^2^*r, a^2^*l (no 3-way interactions)	10	638.73	15.94	−309.4	<0.001
a, a^2^, l, a*r, a*l, r*l, a^2^*r, a^2^*l, a^2^*r*l (no Rain, 3-way interaction uses Age^2^)	10	648.55	25.76	−314.3	<0.001
a, r, l, a*r, a*l, r*l, a^2^*r, a^2^*l, a^2^*r*l (no Age^2^, 3-way interaction uses Age^2^)	10	651.61	28.82	−315.8	<0.001
a, a^2^, r, l, a*r, a*l, r*l, a*r*l (no Age^2^ interactions)	9	655.04	32.24	−318.5	<0.001
a, a^2^, r, l, r*l, a^2^*r, a^2^*l (no Age interactions and no 3-way interaction)	8	661.17	38.37	−322.6	<0.001
a, a^2^, r, l, a*r, a^2^*r (no Low interactions)	7	680.25	57.45	−333.1	<0.001
a, r, l, a*r, a*l, r*l, a*r*l (no Age^2^ and no Age^2^ interactions)	8	681.62	58.82	−332.8	<0.001
a, a^2^, r, a*r, a^2^*r (no Low and no Low interactions)	6	681.87	59.08	−334.9	<0.001
a, a^2^, r, l, a*l, a^2^*l (no Rain interactions)	7	721.75	98.95	−353.9	<0.001
a, a^2^, r, l (no interactions)	5	724.31	101.51	−357.2	<0.001
a^2^, r, l, a*r, a*l, r*l, a^2^*r, a^2^*l, a^2^*r*l (no Age, 3-way interaction uses Age^2^)	10	726.83	104.03	−353.4	<0.001
a^2^, r, l, r*l, a^2^*r, a^2^*l (no Age and no Age interactions, no 3-way interaction)	7	859.00	236.21	−422.5	<0.001
r, l, r*l (no Age, Age^2^_,_ or interactions)	4	923.56	300.77	−457.8	<0.001
a, a^2^, l, a*l, a^2^*l (no Rain or Rain interactions)	6	1054.93	432.13	−521.5	<0.001

Multiple logistic regression was used, grouping on nest, with robust standard errors on 2482 chicks in 590 nests with 233 storms. a = chick age (days) on date of storm (chicks that did not die in a storm were randomly assigned to a storm), a^2^ = age squared, r = rain, l = low temperature, * indicates interaction terms, *k* = number of parameters, AIC = Akaike’s Information Criterion, ΔAIC = the difference between AIC and the lowest AIC, LL = model log-likelihood, AIC weight = the probability that the model is the best model given the data and the set of candidate models. Pseudo *R* ^2^ of the best model was 0.57.

**Table 2:** Partial regression coefficients and robust standard errors for standardized variables in the best model (lowest AIC; Table 1) for the probability that a Magellanic penguin chick died in a storm at Punta Tombo, Argentina, 1983–2010.

Predictor variables	Coefficient	Robust standard error
Age	−3.97	0.60
Age * Rain	−3.48	0.53
Age squared * Rain	3.42	0.54
Age squared	−2.64	0.51
Age squared * Rain * Low	2.06	0.37
Age squared * Low	1.32	0.25
Rain	1.31	0.23
Intercept	−3.24	0.26

We used multiple logistic regression, grouping on nest, with robust standard errors on 2482 chicks in 590 nests with 233 storms. Age = chick age (days) on date of storm (chicks that did not die in a storm were randomly assigned to a storm), Low = low temperature, * indicates interaction terms. Variables were standardized so the coefficient magnitudes indicate their relative strengths.

Chicks in bush nests were more likely to die of exposure during storms than chicks in burrow nests. The best model included only nest type in addition to the weather variables (Table 3). However, nest type, quality, and orientation were not independent of each other. Consistent with the model, our data showed chicks that died in storms came disproportionately from bush nests: 9% of chicks in bush nests died compared to 3% of chicks in burrow nests (*N* = 2123, χ^2^(1) = 19.8, *P*<0.001). Bush nests generally have less cover than burrow nests and 10% of chicks in nests with average and poor cover (<80%) died compared to 6% of chicks in nests with high cover (>80%; *N* = 2482, χ^2^(1) = 19.2, *P*<0.001). The number of chicks that died was independent of nest orientation (*N* = 1063, χ^2^(3) = 4.1, *P* = 0.25).

**Table 3:** Predictor variables and model AIC values for the probability that a Magellanic penguin chick died in a storm at Punta Tombo, Argentina, 1983–2010 for models including nest characteristics.

Predictor variables	*k*	AIC	ΔAIC	LL	AIC weight
Type	9	392.93	0	−187.5	0.57
Type, Quality	11	395.60	2.67	−186.8	0.15
Type, Orientation	12	396.47	3.54	−186.2	0.10
No nest characteristics	8	396.67	3.74	−190.3	0.09
Orientation	11	398.63	5.69	−188.3	0.03
Type, Quality, Orientation	14	398.70	5.77	−185.4	0.03
Quality	10	399.04	6.10	−189.5	0.03
Quality, Orientation	13	400.96	8.03	−187.5	0.01

We used multiple logistic regression, grouping on nest, with robust standard errors on 1063 chicks in 377 nests with 233 storms. We added nest characteristics to the best model of chick age and weather variables (Table 1). Type = nest type (burrow or bush), Quality = nest quality (good, average, or poor; see Materials and Methods), Orientation = nest-entrance orientation (N, E, S, W), *k* = number of parameters, AIC = Akaike’s Information Criterion, ΔAIC = the difference between AIC and the lowest AIC, LL = model log-likelihood, AIC weight = the probability that the model is the best model given the data and the set of candidate models. Pseudo *R* ^2^ of the best model with nest characteristics was 0.57.

The model for chicks in bush nests overestimated the probability of death when rainfall was between 25 and 45 mm. The model captured that most chicks in bush nests that died were of intermediate age, 9 to 23 days old, for 10–20 mm rain. The peak was absent in the model when rainfall was 25–45 mm (Fig. 4A, B). Only 17 of 520 chicks in burrow nests died in storms (Fig. 4C). The model shows a much stronger and younger (5–11 days) intermediate-age peak of mortality for burrow nests than for bush nests. Chicks younger than five days and older than 11 days were better protected in burrow nests than in bush nests (Fig. 4B, D). In both nest types, the data and the model showed that bigger rainstorms killed more chicks and older chicks (Fig. 4).

### Breeding Synchrony and Storm Mortality

**Figure 6:**
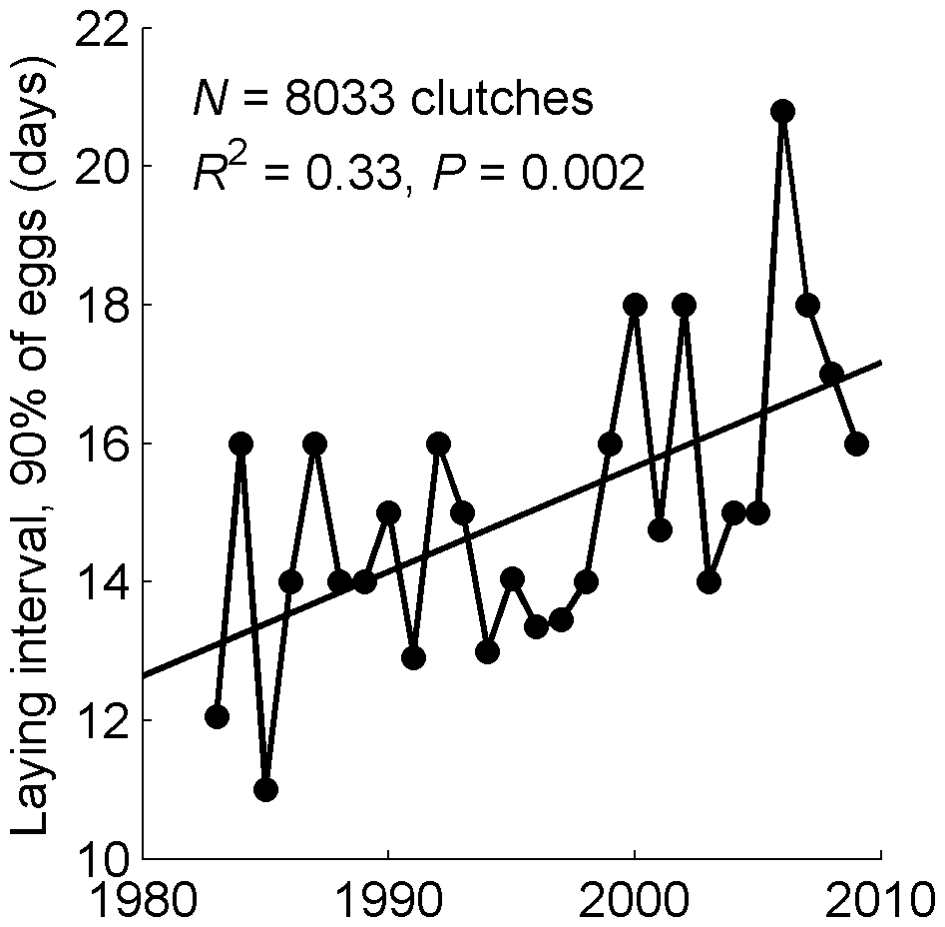
Egg laying in Magellanic penguins has become less synchronous. The laying interval (number of days between the 5^th^ and 95^th^ percentiles of laying dates) of 1^st^ eggs of Magellanic penguins increased between 1983 and 2009 (*F*_1,25_ = 12.2, *P* = 0.002, *R* ^2^ = 0.33, *N* = 8033 clutches).

The synchrony of breeding at Punta Tombo decreased by 4.1 days from 1983 to 2010, or 0.15 days per year (Fig. 6; *F*_1,25_ = 12.2, *P* = 0.002, *R* ^2^ = 0.33). The shortest laying interval was 11 days in 1985 and the longest was 21 days in 2006. Predicted egg laying intervals, based on the regression of laying interval on year, ranged from about 13 days in 1983 to 17 days in 2010. When eggs were laid later, laying was less synchronous (using residuals to control for the increase over time in both laying dates and laying asynchrony; *F*_1,25_ = 19.4, *P* = 0.0002, *R* ^2^ = 0.41). When females weighed less in late September, eggs were laid later than when females weighed more (*F*_1,20_ = 4.6, *P* = 0.045, *R* ^2^ = 0.19).

**Figure 7:**
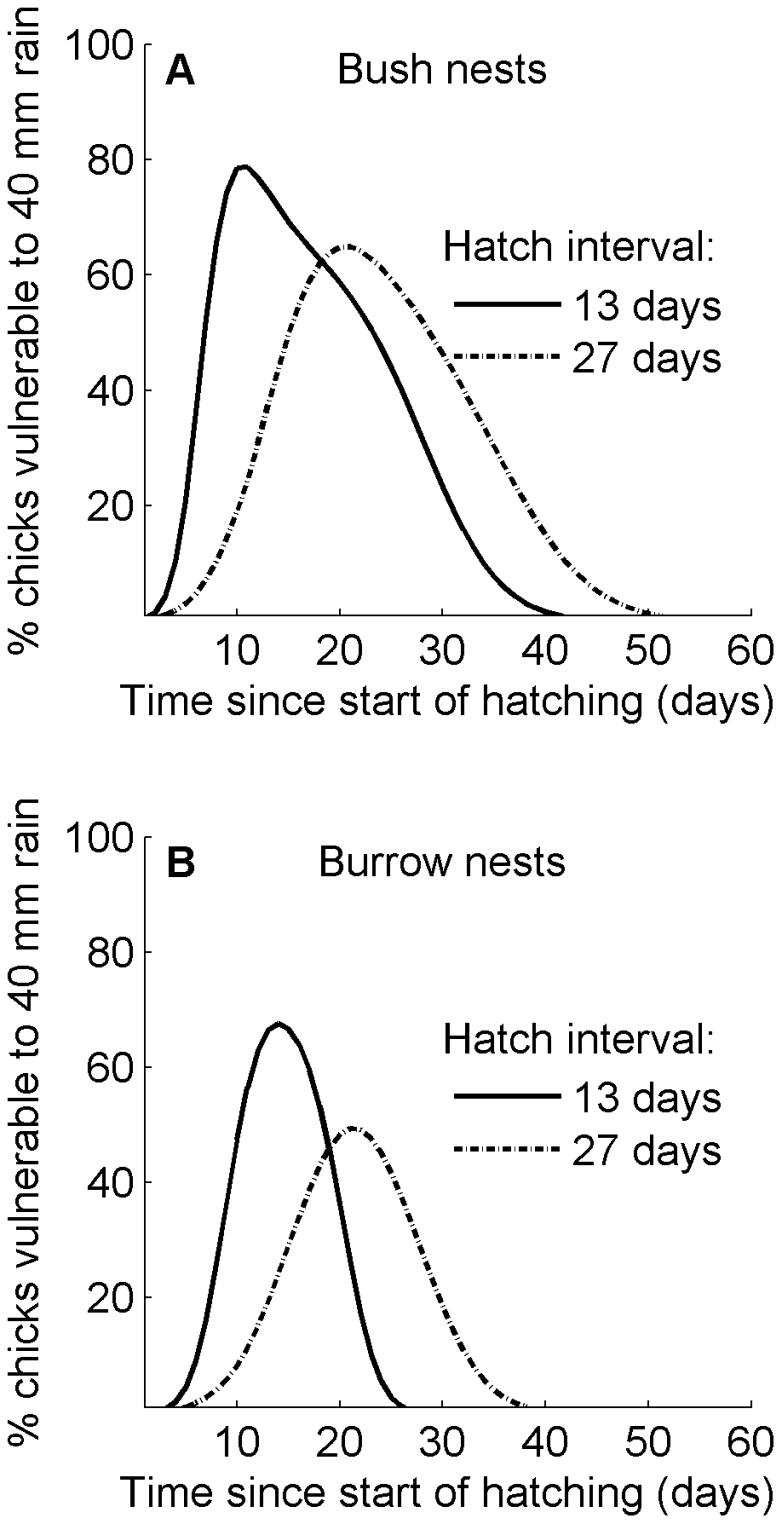
The percentage of Magellanic penguin chicks likely to die in a storm depended on breeding synchrony. The solid lines represent the percentages of chicks likely to die in a storm with 40% of chicks hatched within 13 days. The broken lines represent the percentages of chicks likely to die in the same storm if the hatching interval is 27 days. Both curves assume a normal distribution of hatching dates within the interval and probabilities of dying as shown in Fig. 4 for 40 mm of rain. The curves cross at 19 days. Before that, a storm would kill more chicks if the hatch interval is 13 days; after that, a storm would kill more chicks if the hatch interval is 27 days. (A) Bush nests. (B) Burrow nests.

Storms may occur at any time but as breeding synchrony decreases the time lengthens when some chicks are most vulnerable to storm mortality. When breeding synchrony is lower and hatching occurs over a longer period, fewer chicks are at a vulnerable age when they might die during any one storm (Fig. 7). The period when some chicks in the colony are between 9 and 23 days, however, is longer and therefore the chance of rain is higher. If a storm with 40 mm of rain occurs 10 to 15 days after hatching begins when the hatching interval is 13 days, most chicks are more than 5 days old and all are less than 15 days of age. The storm would likely kill between 70% and 80% of chicks in bush nests. Fewer chicks, about 55–65%, would likely die if the same storm occurred 17 to 27 days after hatching begins (a longer and later peak period) if the egg-laying interval is 27 days (Fig. 7A). Because storm mortality is additive, if 65% of chicks died in a storm during a year of good food availability such as 2008 when only 12% of chicks starved, and predation and other sources of mortality were low, at least ¾ of chicks would likely die. When breeding synchrony is higher and hatching occurs over a 13-day period, at least 10% of chicks in bush nests are vulnerable to being killed in a storm for about 30 days. When synchrony is lower and hatching extends over 27 days instead of 13 days, at least 10% of chicks in bush nests are vulnerable to storm death for about 35 days instead of 30 days (Fig. 7A). Similarly, at least 10% of chicks in burrow nests are vulnerable to being killed in a storm for about 17 days if hatching occurs over 13 days, compared to 22 days if hatching occurs over 27 days (Fig. 7B). Thus, decreased synchrony lengthens the time when a storm is likely to kill some chicks in the colony. Storm date interacts with breeding synchrony to predict how many chicks are killed. If breeding is more synchronous, more chicks die in an early storm, but if breeding is less synchronous, more chicks die in a later storm (Fig. 7).

## Discussion

We show that starvation and predation are the main causes of death in Magellanic penguin chicks at Punta Tombo in most years but rain and heat are important sources of mortality in some years. Rain killed the most chicks in two years out of the 28-year study. Rain and heat are additive to other causes of chick death. We found the number of storms per breeding season increased. Climate models predict that the frequency and intensity of storms will continue to increase. Chicks from about 9–23 days of age, intermediate-age chicks, are more likely to die in a storm than younger or older chicks. Increasing rain, decreasing temperatures, and bush nests compared to burrow nests contribute to chick mortality. The decreasing breeding synchrony of penguins at Punta Tombo interacts with storm timing to influence the number of chicks killed by storms.

### Causes of Death

Most Magellanic penguin chicks died from starvation or predation [46], [47]. Climate variability in the form of increased rainfall and temperature extremes, however, has increased in the last 50 years [6], [7] and kills many chicks in some years. Mortality from rainfall and extreme temperatures is variable among years, but is density independent. High mortality in storms was not compensated by lower starvation or predation rates. Rainfall between mid-October and mid-December explained 78% of the variation in reproductive success from 1997 to 2006 at Punta Tombo [60]. Magellanic penguins at Punta Tombo rarely re-lay eggs so storm mortality is not compensated by replacement clutches. The number of chicks that died from heat and the number that fledged were poorly related, probably because relatively few chicks died from heat and we underestimated heat deaths.

Starvation peaked at 6–8 days of age. When chicks hatch, they can live off the yolk sac for several days, although early feeding in some birds is known to stimulate development of the digestive system and enhances future weight [61], [62]. In domestic chickens some yolk was retained for 16 days, in domestic geese for 7 days, and in domestic ducks for 5 days [63]. The peak in mortality at 6–8 days of age for Magellanic penguins suggests that the yolk reserves for these chicks are depleted by about 7 days of age.

### Mortality from Rainstorms and Heat

We underestimated the effects of storms and heat on reproductive success for several reasons. First, we could not determine the cause of death for some chicks that may have died of exposure or heat. Second, temperature is not the only factor causing chicks to die from heat. Wind speeds and directions that increase evaporation and water stress, as well as poor nest cover, contribute to heat deaths. Third, we did not include eggs, which can be killed by rain [13], [15], [57], [64]. Even storms that do not cause death are energetically costly to seabird chicks. A modeling study on Adélie penguins (*Pygoscelis adeliae*) showed that when just 10% of a chick’s surface area was wet, growth rate decreased, preventing the chick from reaching the fledging mass needed for successful recruitment [65].

Starvation and weather likely interact to increase mortality as chicks that are fed better have more energy to maintain their body temperatures. Insufficient food reduces a chick’s thermoregulatory ability [66]. A starving chick is more likely to die in a storm or extreme temperature than a well-fed chick, and a chick waiting for a meal is likely to last longer if there is not a storm or extreme temperature. Insufficient food should increase a chick’s susceptibility to heat because a small chick’s sole source of water is its food. Chicks that are not fed thus have reduced ability to use evaporative cooling and may be more likely to stay in an exposed nest waiting to be fed. A combination of heat and lack of food may kill more chicks than heat alone or starvation alone. We could not tease apart these variables. Finally, storms and heat may increase starvation and predation when adults feed chicks less frequently because of reduced foraging efficiency or nest attendance during extreme weather [19], [20], [21], [24].

November and December, when penguin chicks are most vulnerable to rain, tend to be wetter in the Punta Tombo region during El Niño years than other years, and Novembers are wetter preceding and following La Niña years [67]. Although climate models disagree whether El Niño events will worsen in the 21^st^ century, El Niño years were stronger in the late 20^th^ century compared to the previous 700 years [68] and models show that El Niño is likely to respond to increasing CO_2_ following the 21^st^ century if not sooner [69].

### Age, Rainfall, Low Temperature, and Nest Type Affect Mortality

Storm timing affects population dynamics in species ranging from plants [70] to mammals [71]. Chick age during the storm, determined by storm timing, was the most important variable in our models. Penguin chicks at hatching depend on brooding by parents to stay dry and warm, but as chicks age, their down and juvenile plumage grows, surface-to-volume ratio decreases, metabolic rate increases and their ability to thermoregulate improves [72], [73].

Chicks younger than about 5 days are small enough to fit into the parent’s brood patch and be protected from the weather. Penguin chicks older than about 5 days of age can be too large to fit completely into the brood patch and some do not seek shelter in a storm (Boersma, pers. obs.). Larger gentoo (*Pygoscelis papua*) and chinstrap (*P. antarctica*) penguin chicks also could not be completely brooded during storms [73]. Intermediate-age chicks are too young to thermoregulate sufficiently to survive storms because their juvenile plumage is not long enough to offer protection. Gentoo, chinstrap, and king (*Aptenodytes patagonicus*) penguin chicks can thermoregulate effectively by 15 days of age when they are dry [72], [73]. When a chick’s down gets wet, it loses the insulating air spaces, and heat loss increases. The rate of conductive heat loss through wet down is increased by the higher thermal conductivity of water compared to that of air [65]. Although a chick between 15 and 30 days of age can thermoregulate well when dry, it may be unable to maintain its body temperature when its down is wet [12].

The two storms that killed >50% of chicks both occurred in early December, when chicks were zero to 19 days of age (1999) or eight to 23 days of age (1991). The latest storm that killed chicks was 28 December, with 43 mm of rain that caused flooding of many nests. Heavier rain (47–134 mm) that occurred later did not kill chicks because chicks were old enough to have waterproof plumage and thermoregulate well.

Heavier rain and lower minimum temperature increased chick mortality in storms. Climate models predict that a storm of 40 mm that was expected to occur at Punta Tombo every 20 years in the late 20^th^ century, will occur every 7–15 years by 2081–2100 [7]. The increased frequency of heavy rain means that reproductive failures will occur more often. Minimum air temperatures at Trelew airport decreased over the same period that rain became heavier [32], [35]. If heavy rain coincides with lower minimum temperatures, more chicks will die, especially if the storms occur when chicks are under 30 days of age.

Good-quality nests protect Magellanic penguin chicks from predators and heat [24] and we showed that well-covered burrow nests also helped protect Magellanic penguin chicks from storms. Nest-site characteristics affected chick mortality from storms in several seabird species, including European shags [40], little penguins (*Eudyptula minor*) [64], African penguins (*Spheniscus demersus*) [74], and manx shearwaters (*Puffinus puffinus*) [15]. After southern rockhopper penguin (*Eudyptes chrysocome*) chicks left their nests to form crèches, vegetation cover still helped protect them from storms [12]. Nest orientation had the least effect on chick mortality of the nest characteristics we tested. Rain seldom falls vertically at Punta Tombo during storms because of high winds. Although the coldest winds typically come from the south, rain can come from any direction. Orientation is also unlikely to have much impact on flooding because the direction of water flow is likely more dependent on ground slope than on burrow orientation or rain direction. Other characteristics, such as burrow depth, nest-opening size, and landscape features may be as important as orientation.

For penguins that breed in hot, dry environments, shade may be more important than protection from rain in most years [24]. Reproductive success is higher in burrows than in open nests for *Spheniscus* penguins and high air temperatures can stress or kill adults and chicks in various species [23], [24], [75], [76]. Burrows provide protection from sun and aerial predators so are favored in most years. However, burrows may collapse during or after heavy rain [22], [54], or they may hold water because of the increased clay content of soil suitable for burrowing [54], making them risky in years of heavy rain. As rain becomes more frequent and intense, burrows may become more or less preferred depending on their tendency to collapse or flood. Humboldt penguins in central Chile frequently abandon flooded nests in the autumn breeding season, causing autumn to be less productive than the spring nesting season. El Niño events bring particularly heavy rains and can affect the following breeding season because nests remain flooded [77]. Finding a nest site that is ideal for all weather will be harder for penguins; phenotypic plasticity (in terms of nest-site selection) may not be able to adjust for more frequent extreme events [4].

### Breeding Synchrony and Storm Mortality

The decreasing breeding synchrony of Magellanic penguins at Punta Tombo lengthens the period when a proportion of chicks is vulnerable to storms. The increasing frequency of large storms also lengthens the period when a proportion of chicks is vulnerable because large storms kill older chicks. Timing of storms will continue to be a major factor in chick mortality, but storms 2–3 weeks later in the season will have a greater impact if breeding synchrony continues to decrease. The simulation in Fig. 7 used the same rainfall for past and potential breeding synchrony. If we had used a larger storm, more likely in the future, for the 27-day hatching interval, the peaks would be more similar in height, but the width of the 27-day curve would be even wider. Hence the interaction of increasing rain and decreasing breeding synchrony will kill more chicks.

### Effects of Climate on Recruitment and Population Trends

For birds and mammals, which physiologically maintain their body temperatures at relatively constant values, effects of climate change are often indirect, through changes to food webs [78], [79], [80], [81]. The direct effect of climate change through storms on chick mortality and reproductive success is probably more important than currently recognized. When large numbers of chicks die in storms, few chicks fledge and future recruitment from that cohort will be low. In most years, food availability determines reproductive success of the colony and overwinter survival of juveniles, and therefore recruitment from the cohort. In years with heavy rain when chicks are young, future recruitment is driven by weather and is decoupled from food availability.

Population trends in long-lived seabirds are affected by reproductive output as well as adult survival [82], [83], [84]. Although a single reproductive failure due to a storm may not affect population dynamics in long-lived species, the increasing frequency of reproductive failures due to more frequent and intense storms from climate change is likely to reduce recruitment and population size over the long term. Increased frequency of reproductive failure and juvenile, as opposed to adult mortality, theoretically select for reduced reproductive effort [4]. In addition, increased variation in demographic parameters can lead to decreased population growth rates even if the mean values of the parameters do not change [3], [85]. As large storms become increasingly frequent, there is the potential to overwhelm the population’s ability to recover from each storm. Weather extremes can affect populations more strongly than average climate [4], [86], and multiple perturbations that occur within the generation time of a population can lead to community-composition changes and local extinctions [87].

## Conclusions

Magellanic penguins are still abundant but the number of penguins breeding at Punta Tombo has declined more than 20% since 1987 [30]. There are a number of factors contributing to this decline such as petroleum pollution [88], [89]. However, climate change will likely make weather an increasingly important factor in chick mortality, and deaths due to weather are additive to starvation and predation. In addition, Magellanic penguins at Punta Tombo are laying eggs later [90] and swimming farther to forage for their chicks [60], both factors that reduce reproductive success. Over a 28 year period storms killed only 8% of Magellanic penguin chicks at Punta Tombo. However one storm killed 50% of a cohort of chicks. These events appear to be increasing as climate changes, further stressing the population.

Increasing trends in extreme rainfall are predicted over most of the breeding range of Magellanic penguins [7], [8] so increasing rain will likely increase the frequency of breeding failures throughout the species’ range. Of the 18 penguin species, five are endangered and six are vulnerable [28]. Seabirds are among the most threatened groups of birds and their status has rapidly gotten worse, with many of the species of most concern found in the southern hemisphere [91]. Other seabirds’ breeding ranges overlap that of Magellanic penguins, including at least 16 species along the coast of Argentina [92], 25 in the Falkland/Malvinas Islands [93], and 19 in southern Chile [94], [95]. These species are also likely to be negatively impacted by climate change, a density-independent factor that can remove most of a cohort. Increasing storminess bodes ill not only for Magellanic penguins but for many other species including humans.
